# Supplementary material for: The Lipid Paradox Among Acute Ischemic Stroke Patients-A Retrospective Study of Outcomes and Complications
Source: Medicina (Kaunas). 2019 Aug 13;55(8):475. doi: 10.3390/medicina55080475 (PMC6723697; doi:10.3390/medicina55080475)
Supplement: Supplementary file 1 [file medicina-55-00475-s001.zip › Supplementary Material.docx]

**SUPPLEMENTAL MATERIAL**

**Supplementary Table 1: ICD-9-CM codes used in this analysis**

| **Condition** | **ICD-9 CM Codes** |
| --- | --- |
| Hypercholesterolemia | 272.0, 272.1, 272.2 |
| Obesity | 278.00, 278.01, 278.02 |
| Hypertension | 401-405 |
| Diabetes Mellites | 249.00, 250 |
| Alcohol abuse/dependent | V11.3, 303, 305.0 |
| Tobacco Current/Past use | V15.82, 305.1 |
| Drug abuse | 304, 305.2-305.9 |
| AIDS | 042, V08 |
| Atrial fibrillation | 427.31 |
| Hemorrhagic conversion | 430, 431 |
| Stroke Associate Pneumonia | 481, 482, 486, 997.31, 997.32 |
| Epilepsy | 345, 780.39 |
| Gastrointestinal bleeding | 530.04, 531.00, 531.01, 531.40, 531.41, 531.10, 531.20, 531.21, 531.50, 531.51, 531.60, 531.61, 532.00, 532.01, 532.40, 532.41, 532.10, 532.11, 532.20, 532.21, 532.50, 532.51, 532.60, 532.61  533.00, 533.01, 533.40, 533.41, 533.50, 533.10, 533.11, 533.20, 533.21, 533.50, 533.51, 533.60, 533.61, 534.10, 534.11, 534.20, 534.21, 534.50, 534.60, 535.01, 535.11, 535.41, 535.51, 535.61, 534.61, 578.0 |
| Atrial fibrillation | 427.31 |
| Use of antiplatelet current use | V58.63 |
| Use of anticoagulant current use | V58.61 |
| Use of aspirin | V58.66 |
| Use of IV tPA during same hospitalization or in a different institution within the last 24 hours prior to admission to facility | 99.10, V45.88 |
| Helicobacter Pylori | 041.86 |
| Gastrostomy | 431.1, 431.9 |
| Helicobacter Pylori | 041.86 |
| Nasogastric tube | 96.07 |
| Endotracheal intubation | 96.04 |
| Non-invasive Mechanical Ventilation | 93.90 |
| Invasive Mechanical Ventilation | 96.70-96.72 |

**Supplementary Table 2: Deyo’s modification of Charlson’s co-morbidity index (CCI)**

| **Condition** | **ICD-9-CM Codes** | **Charlson Score** |
| --- | --- | --- |
| Myocardial infarction | 410 – 410.9 | 1 |
| Congestive heart failure | 428 – 428.9 | 1 |
| Peripheral vascular disease | 433.9, 441 – 441.9, 785.4, V43.4 | 1 |
| Cerebrovascular disease | 430 – 438 | 1 |
| Dementia | 290 – 290.9 | 1 |
| Chronic pulmonary disease | 490 – 496, 500 – 505, 506.4 | 1 |
| Rheumatologic disease | 710.0, 710.1, 710.4, 714.0 – 714.2, 714.81, 725 | 1 |
| Peptic ulcer disease | 531 – 534.9 | 1 |
| Mild liver disease | 571.2, 571.5, 571.6, 571.4 –571.49 | 1 |
| Diabetes | 250 – 250.3, 250.7 | 1 |
| Diabetes with chronic complications | 250.4 – 250.6 | 2 |
| Hemiplegia or paraplegia | 344.1, 342 – 342.9 | 2 |
| Renal disease | 582 – 582.9, 583 – 583.7, 585, 586, 588 – 588.9 | 2 |
| Any malignancy including leukemia and lymphoma | 140-172.9, 174-195.8, 200-208.9 | 2 |
| Moderate or severe liver disease | 572.2 – 572.8 | 3 |
| Metastatic solid tumor | 196-199.1 | 6 |
| AIDS | 042 – 044.9 | 6 |
